# Influenza Virus-Induced Novel miRNAs Regulate the STAT Pathway

**DOI:** 10.3390/v13060967

**Published:** 2021-05-23

**Authors:** Sreekumar Othumpangat, Donald H. Beezhold, Christina M. Umbright, John D. Noti

**Affiliations:** 1 Allergy and Clinical Immunology Branch, Health Effects Laboratory Division, Centers for Disease Control and Prevention, National Institute for Occupational Safety and Health, Morgantown, WV 26505, USA; zec1@cdc.gov (D.H.B.); ivr2@cdc.gov (J.D.N.); 2 Toxicology and Molecular Biology Branch, Health Effects Laboratory Division, Centers for Disease Control and Prevention, National Institute for Occupational Safety and Health, Morgantown, WV 26505, USA; cumbright@cdc.gov

**Keywords:** novel miRNA, epithelial cells, STAT pathway, influenza virus

## Abstract

MicroRNAs (miRNAs) are essential regulators of gene expression in humans and can control pathogenesis and host–virus interactions. Notably, the role of specific host miRNAs during influenza virus infections are still ill-defined. The central goal of this study was to identify novel miRNAs and their target genes in response to influenza virus infections in airway epithelium. Human airway epithelial cells exposed to influenza A virus (IAV) induced several novel miRNAs that were identified using next-generation sequencing (NGS) and their target genes by biochemical methods. NGS analysis predicted forty-two RNA sequences as possible miRNAs based on computational algorithms. The expression patterns of these putative miRNAs were further confirmed using RT-PCR in human bronchial epithelial cells exposed to H1N1, H9N1(1P10), and H9N1 (1WF10) strains of influenza virus. A time-course study showed significant downregulation of put-miR-34 in H1N1 and put-miR-35 in H9N1(1P10)-infected cells, which is consistent with the NGS data. Additionally, put-miR-34 and put-miR-35 showed a high fold enrichment in an argonaute-immunoprecipitation assay compared to the controls, indicating their ability to form a complex with argonaute protein and RNA-induced silencing complex (RISC), which is a typical mode of action found with miRNAs. Our earlier studies have shown that the replication and survival of influenza virus is modulated by certain transcription factors such as NF-ĸB. To identify the target(s) of these putative miRNAs, we screened 84 transcription factors that have a role in viral pathogenesis. Cells transfected with mimic of the put-miR-34 showed a significant decrease in the expression of Signal Transducers and Activators of Transcription 3 (STAT3), whereas the inhibitor of put-miR-34 showed a significant increase in STAT3 expression and its phosphorylation. In addition, put-miR-34 had 76% homology to the untranslated region of STAT3. NGS and PCR array data submitted to the Gene Ontology project also predicted the role of transcription factors modulated by put-miR-34. Our data suggest that put-miR-34 may be a good target for antiviral therapy.

## 1. Introduction

MicroRNAs (miRNAs) belong to a class of endogenous non-coding small RNA molecules that regulate gene expression. In the past decade, great effort has been made to understand the functional role of miRNAs in various biological processes, including organ development, cell differentiation, cell cycle, and apoptosis [1,2,3]. The biosynthesis of miRNA involves a series of steps as described earlier [3]. Most single-stranded miRNAs are loaded onto argonaute (AGO), positioned at the core of RNA-induced silencing complex (RISC) to target coding RNAs by sequence complementarity. As a result, target gene expression is repressed through translational inhibition and RNA degradation [2,3]. Next-generation sequencing (NGS, deep sequencing) has provided a powerful tool to identify differentially expressed miRNAs, especially low-abundance ones, under conditions of physiological perturbation.

Influenza A virus (IAV) infection induces several miRNAs that are already published or available in the miRBase release 22 (www.mirbase.org (accessed on 1 October 2020), but little information is available regarding on miRNAs specifically produced following exposure to IAV. Therefore, here, we attempted to identify novel miRNAs that were elicited upon exposure to IAV in human bronchial epithelial cells (HBEpCs) using NGS. Wang et al., also using NGS, studied the avian influenza virus, and a total of 271 miRNAs were identified, of which 121 miRNAs were differentially expressed and one was a potentially novel miRNA [4]. Another study reported the presence of putative miRNAs associated with H1N1 that have a role in host–virus interactions [5]. Exploring miRNAs that can degrade both IAV RNAs and ease the virus-induced inflammation is very important to control the viral replication and immune responses. In addition, establishing the role of miRNAs in signaling pathways associated with IAV infection is essential to fully illustrate their probable use in disease diagnosis, prognosis, and ultimately for the treatment of disease. Our previous studies have shown that transcription factor Nuclear factor κB (NF-κB) and its inhibitor, NFKBIB, play a crucial role in regulating the survival and propagation of IAV [6,7]. The NF-κB family of proteins was initially characterized as a group of transcription factors critical in the immune response to pathogens and other foreign bodies [8,9]. Subsequently, these proteins have been found to regulate the expression of a variety of genes responsible for diverse biological processes, including cell proliferation, migration, survival, and stress response. Studies have also shown that transcription factors that control the transcription of genes are essential regulators in viral replication [10].

Transcription factors have the function of either stabilizing (as an activator) or blocking (as a repressor) the binding of RNA polymerase to DNA and result in the direct control of gene expression. Among the transcription factors, Signal Transducers and Activators of Transcription (STAT) family members are known to regulate several viruses, such as, Epstein–Barr virus infection (STAT1), dengue virus (STAT2) [11,12], and Respiratory syncytial virus (STAT3) [13]. Several DNA and RNA viruses are also known to regulate STAT3. STAT3′s role has been well characterized in the pathogenesis of viral infections. In addition, STAT3 and the NF-κB signaling pathway play a critical role in the anti-inflammatory function [14]. In contrast, IAV nonstructural protein 1 (NS1) impedes STAT3 phosphorylation in infected cells [15]. STAT3 becomes hyperactivated or inactivated in viral disease, exhibiting a tightly regulated STAT3 function. As such, STAT3 is vital for many important cellular processes that include WNT, VEGF, TLRs, TGFβ, signal transduction, PDGF, Notch, MAP Kinases, JNK, and JAK/STAT pathways [16]. In this study, we sought to identify novel miRNAs that are specifically produced upon exposure to IAV and their role(s) in signaling pathways including transcription factors, which may provide a unique nature of the miRNAs and their role in controlling the multiplication and survival of the virus in human bronchial epithelium.

## 2. Materials and Methods

### 2.1. Cell Culture

Primary human bronchial epithelial cells (HBEpCs) and small airway epithelial cells (SAEpCs) were purchased from PromoCell GmbH (Heidelberg, Germany) and sub-cultured in media supplemented with growth factors as recommended by the supplier. Madin–Darby Canine Kidney (MDCK) cells were cultured in Eagle’s Minimum Essential Medium (ATCC, Manassas, VA, USA) with 10% fetal bovine serum, and appropriate antibiotics. MDCK cells were used for the propagation of IAV H1N1 (A/WSN/33), H9N1 (1P10), and H9N1 (1WF10). IAV H1N1 (A/WSN/33) was obtained from Prof. Robert A. Lamb (Northwestern University, Chicago, IL, USA), and H9N1 strains were obtained from Daniel Perez (Georgia University, GA, USA). Cultivation of the virus was carried out as described earlier [6,7,17].

### 2.2. Viral Infections

All infections of HBEpCs and SAEpCs were performed in 6-well plates (Corning, NY, USA) at different multiplicities of infection (MOI). All experiments were done in duplicates, and the experiments were repeated on 3 different days. Control cells were mock infected. Prior to infection, the cells were rinsed with PBS; then, virus diluted in Modified Hanks Buffer Saline Solution was added to each well. After a 45-min incubation, excess virus was rinsed off using cold PBS. Fresh F12 media containing 1 µg/mL of TPCK (L-(tosylamido-**2**-phenyl) ethyl chloromethyl ketone) modified-trypsin (Sigma-Aldrich, St Louis, MO, USA) was added to H9N1 strains and medium lacking TPCK was added to the H1N1strain. Both plates were incubated at 37 °C and 5% CO_2_. Cells were harvested at different time intervals as described in methods.

### 2.3. Next-Generation Sequencing (NGS)

NGS analysis was conducted on total RNA isolated from SAEpCs infected with IAV H1N1 and both H9N1 strains 1WF10 and 1P10 to identify differentially expressed host miRNAs, both known in miRbase release 22 and unknown miRNAs (putative miRNA). SAEpCs were infected with IAV for 3 h as described earlier (4). Total RNA including miRNA was isolated from infected and uninfected cells using the miRNeasy kit (Qiagen, Germantown, MD, USA), and the quality of the total RNA was assessed by Agilent 2100 Bioanalyzer and Agilent RNA 6000 Nano Reagents (Santa Clara, CA, USA). Then, the isolated total RNA was sent to Exiqon (Aarhus, Denmark) for NGS analysis and the identification of differentially expressed miRNAs.

### 2.4. NGS Data Analysis

The amplification efficiency was calculated using algorithms similar to the LinRegPCR [18,19] a standalone software (LinRegPCR ver 11.0; http://LinRegPCR.nl (accessed on 22 May 2021)). All assays were inspected for distinct melting curves, and the Tm was checked to be within known specifications for the assay. Cq was calculated as the 2nd derivative. Using NormFinder [20], the best normalizer was found to be the average of assays detected in all samples. All data were normalized to the average of assays detected in all samples (average—assay Cq). When comparing the two groups, a few miRNAs were found to be differentially expressed when using a *p*-value cutoff of 0.05. Two of these passed the Benjamini–Hochberg correction [21] for multiple testing at a significance level of 0.05. Gene Ontology (GO) Enrichment Analysis was used to describe genes, gene products, and their attributes using GO terms. This enables the functional interpretation of experimental data using GO terms through enrichment analysis.

### 2.5. MicroRNA Analysis

A time course study (0–8 h) on putative miRNA expression in cells exposed to IAV was carried out as described previously [7]. Total RNA, including miRNA, was isolated from HBEpCs infected with IAV H1N1 and H9N1 (1P10) or mock-infected cells using an miRNeasy kit (Qiagen, Germantown, MD, USA) following the protocol of the supplier. The quality of the total RNA was verified by an Agilent 2100 Bioanalyzer (Santa Clara, CA, USA) profile following the procedure as suggested by the manufacturer. RT-PCRs were performed to determine the expression of selected miRNAs (put-miR-31, 34, 35) on cDNA synthesized from total RNAs, including miRNAs, isolated from HBEpCs. Specific TaqMan primers were synthesized (Thermo Fisher Scientific, Carlsbad, CA, USA) and used with the TaqMan assay as described earlier [6]. Fold changes of miRNA were calculated using the ddCt method, where Ct is the threshold cycle to detect fluorescence. The data were normalized to control, and the fold change of miRNAs was calculated against control miRNA (U6 snRNA).

### 2.6. Transfection Studies

Putative miRNAs were further validated following HBEpCs exposed to H1N1 and H9N1 for 3 h, and the miRNA was extracted and analyzed for the expression of predicted putative miRNAs. Total RNA was isolated, and 10 ng RNA was reverse transcribed in 10 μL reactions using the miRCURY LNA™ Universal RT microRNA PCR, polyadenylation, and cDNA synthesis kit (Exiqon, Aarhus, Denmark). cDNA was diluted 100 x and assayed in 10 µL PCR reactions according to the protocol for miRCURY LNA™ Universal RT microRNA PCR; each microRNA was assayed once by qPCR on the microRNA Ready-to-Use PCR, Custom Pick and Mix Panel using ExiLENT SYBR^®^ Green (Exiqon, Aarhus, Denmark) master mix. Negative controls excluding template from the reverse transcription reaction were performed and profiled similarly to the samples. Amplification was performed in a LightCycler^®^ 480 Real-Time PCR System (Roche, Indianapolis, IN, USA) in 384-well plates, and amplification curves were analyzed using the Roche LC software, both for determination of Cq (by the 2nd derivative method) and for melting curve analysis.

In another set of experiments, HBEpCs were transfected with put-miR-34 inhibitor oligonucleotide or a put-miR-34 mimic oligonucleotide (Thermo Fisher Scientific, Carlsbad, CA, USA) using the lipid-based Lipofectamine 2000 reagent diluted in Opti-MEM-I reduced serum medium (Life Technologies, Carlsbad, CA, USA) according to the supplier’s protocol. Briefly, HBEpCs were grown to 80% confluence in 6-well plates (Corning), and the transfection complexes were directly applied to the cells as described earlier [7]. As a negative control, cells were transfected with the same concentrations of scrambled oligonucleotides (Thermo Fisher Scientific). Following the incubation, cells were harvested and used for RNA extraction using the RNeasy kit (Qiagen). For the transfection factor PCR array (Themofisher Scientific), the plates were run in duplicates only.

### 2.7. Argonaute (AGO) Immunoprecipitation

AGO immunoprecipitates were isolated from HBEpCs transfected with mimics of put-miR-31, 34, and 35. RNA isolated from the immunoprecipitates were analyzed as described [22]. Briefly, cells were grown to confluency in 6-well plates (Corning). After 24 h of plating, cells were transfected with the mimics of put-miRNAs or a scrambled mimic miRNA (control) using lipofectamine 2000. After 24–36 h of transfection, cells were scrapped off by in cold PBS and lysed using the lysis buffer provided by the supplier (IP kit, Active motif, Carlsbad, CA, USA) following the manufacture’s instruction. Then, RNA was isolated followed by cDNA synthesis. Then, the cDNA was used as a template for the fold enrichment and identification of ago-associated miRNAs by RT-PCR, using specific Taqman primers for put-miR-31, 34, and 35 (Thermo Fisher Scientific). The data were normalized to control, and fold enrichment of miRNAs was calculated as described in supplier’s protocol.

### 2.8. Western Immunoblotting

HBEpCs were transfected with put-miR-34 mimic or inhibitor for 24 h and then mock infected or infected with H1N1 of MOI of 0.5 and 1.0. After 18 h post infection (p.i.), cells were lysed in 100 µL of radioimmunoprecipitation assay (RIPA) buffer (Thermo Fisher Scientific) containing a protease inhibitor cocktail mixture (Thermo Fisher Scientific). Thirty micrograms of protein were solubilized in protein sample buffer and subjected to electrophoresis on 10% SDS-polyacrylamide gels (SDS-PAGE). Separated proteins were transferred to a nitrocellulose membrane (Bio-Rad). Membranes were blocked using Odyssey Blocking Buffer (LI-COR Biosciences, Lincoln, NE, USA) for 1 h at room temperature and subjected to Western analysis with rabbit polyclonal anti-STAT3, anti-pSTAT3 (Abcam, Cambridge, MA, USA), and mouse monoclonal anti-GAPDH (Abcam, MA) antibodies. Appropriate mouse and rabbit IRDye 680 or 800 secondary antibodies (LI-COR Biosciences) were used. Near-infrared fluorescence detection was performed on the Odyssey Imaging System (LI-COR Biosciences, Lincoln, NE, USA), and the fluorescent signal intensities of the individual bands were determined and normalized to GAPDH. All quantitation was done using the manufacturer’s software.

### 2.9. Statistical Analysis

One-way analysis of variance (ANOVA) was used to analyze time course studies as well as studies of mimic and inhibitor on put-miR-34 expression, and post-hoc pairwise multiple comparisons between means were performed using the Holm–Sidak method. Student’s *t*-test was used for the analysis on putative miRNA pair-wise comparison to the controls. NGS data was also subjected to the Benjamini–Hochberg correction [21] for multiple testing at a significance level of 0.05. A *p*-value < 0.05 was considered statistically significant using Sigma Stat version 11.0 for Windows (Systat Software, Chicago, IL, USA).

## 3. Results

### 3.1. Influenza Virus Infection Induce Novel Putative miRNAs

**Figure 1:**
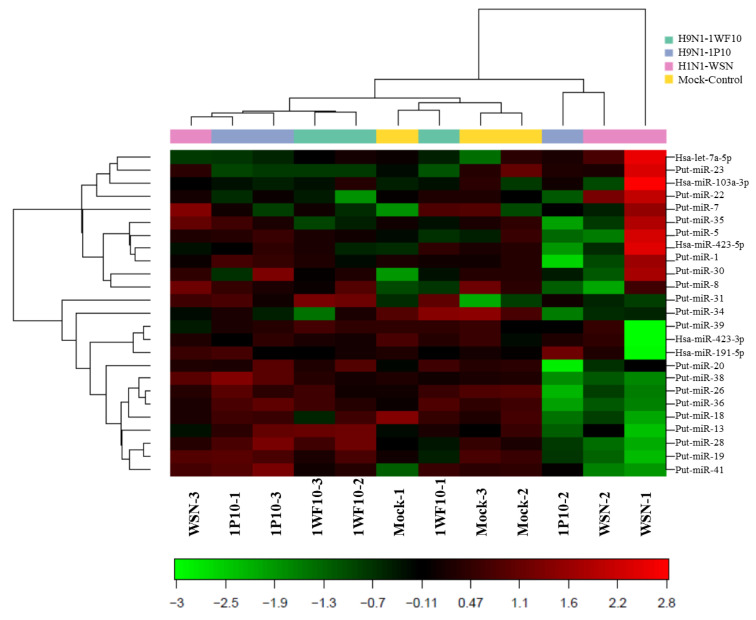
Heatmap with two-way hierarchical clustering of samples and miRNA generated from the NGS analysis of SAEpCs infected with IAV and the control. The clustering was performed on all samples and the top 50 miRNAs with highest coefficient of variation (% CV) based on tag per million (TPM) counts. Each row represents miRNA and each column represent sample. The color of each point represents the relative expression level of an miRNA across all samples. In the color scale shown at the bottom, red represents expression level above the mean, and green represents an expression level below the mean.

To identify IAV-induced novel miRNAs, SAEpCs were infected with H1N1 (WSN), H9N1 (IP10), and H9N1 (1WF10) strains, and the isolated RNA from the infected cells was subjected to NGS. NGS analysis along with the corresponding computational algorithms revealed differential expression of several unknown (putative) as well as previously identified miRNAs. The heatmap of NGS analysis (Figure 1) shows the expression of known (miRBase 22) and putative miRNAs that were upregulated (red) or downregulated (green) in IAV-infected SAEpCs. Then, attention was focused on those putative miRNAs and their potential role and possible targeted mRNAs in human airway epithelial cells. Table 1 shows the sequence of 42 putative miRNAs (as predicted by the NGS data and folding potential based on miRPara analysis), position in chromosome, whether on plus or minus strand, and the predicted sequence using the hg19 genome assembly.

### 3.2. Confirmatory Studies of Selected Putative miRNAs

In order to confirm and evaluate the NGS-predicted miRNAs, we generated individual primer pair sequences for the 42 putative miRNAs and evaluated the miRNA expression levels in HBEpCs infected with H1N1 and H9N1(1P10, 1WF10) strains using RT-PCR. Table 2 shows the fold changes in expression of the putative miRNAs in order based on their *p*-values following H1N1 infection. Interestingly, not all 42 predicted miRNAs were detected. Only 27 of the 42 predicted miRNAs exhibited a fold change, and of those, only 20 miRNAs showed a measurable *p*-value; this is due to missing counts in at least one of the triplicate samples. Put-miR-34 showed a −2.72-fold downregulation (*p* < 0.05) in H1N1-infected cells compared to the mock-infected control cells. The false discovery rate (FDR) analysis showed a lower value for put-miR-34 (0.375) compared to all other miRNAs that had a higher FDR value (≥0.675). Similarly, cells infected with influenza strain H9N1(1P10) showed an increase in put-miR-31 (2.2-fold) expression (*p* ≤ 0.05), while put-miR-34 showed a decreased expression (−3.0-fold) (Table 3). Consistent with the results of infected H1N1, infected H9N1(1P10) also did not reveal all 42 putative miRNA sequences predicted by the NGS study; only 27 exhibited a fold change, and 21 miRNAs showed a *p*-value, due to the lack of counts in at least one of the samples. H9N1 (1WF10)-infected cells showed a difference in the expression of put-miR-31 and put-miR-35 compared to mock-infected cells. Cells infected with H9N1(1WF10) showed a −1.5-fold reduction in put-miR-35 expression, whereas the other put-miRNAs were similar to strain H9N1(IP10) in their expression (data not shown). Since all three strains of influenza virus showed a differential expression of put-miR-31, put-miR-34, and put-miR-35, these three miRNAs were studied further to understand their role in IAV infection and replication in human airway epithelium.

**Figure 2:**
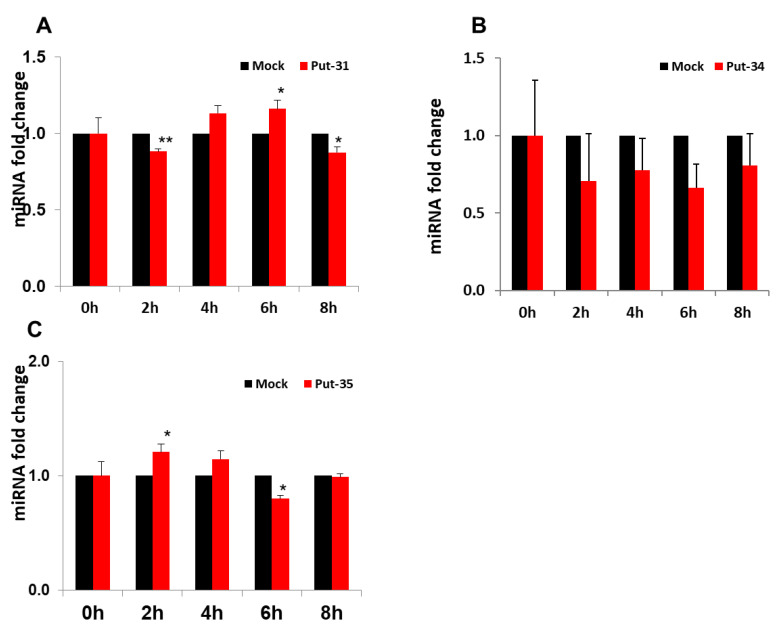
Validation of the putative miRNAs in HBEpCs infected with H1N1 virus. (**A**) Relative expression of put-miR-31 in comparison to the uninfected control cells, using RT-PCR. (**B**) Relative expression of put-miR-34 in comparison to the uninfected control cells, using RT-PCR. (**C**) Relative expression of put-miR-35 in comparison to the uninfected control cells, using RT-PCR. miRNA expression was normalized to the expression of U6 (shown is the mean ± error). HBEpC were infected with 1 MOI of H1N1 and samples were taken at different time intervals. The results are representative of more than three independent experiments. The results from controls were significantly different from the H1N1-infected cells by Student’s *t* test (** *p* < 0.01, * *p* < 0.05). put-31 = put-miR-31; put-34 = put-miR-34; put-35 = put-miR-35.

**Figure 3:**
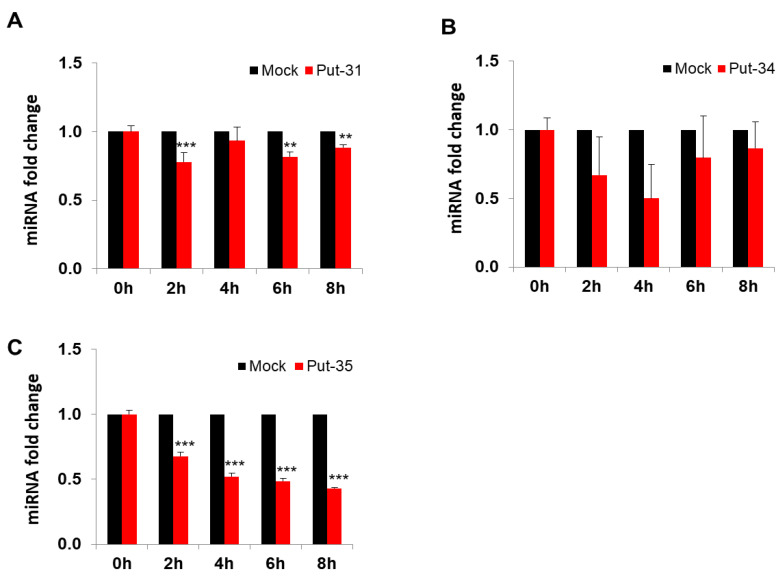
Validation of the putative miRNAs in HBEpCs infected with H9N1 (1P10) virus. (**A**) Relative expression of put-miR-31 in comparison to the uninfected control cells, using RT-PCR. (**B**) Relative expression of put-miR-34 in comparison to the uninfected control cells, using RT-PCR. (**C**) Relative expression of put-miR-35 in comparison to the uninfected control cells, using RT-PCR. miRNA expression was normalized to the expression of U6 (shown is the mean ± error). HBEpCs were infected with 1 MOI of H9N1(1P10), and samples were taken at different time intervals. The results are representative of more than three independent experiments. The results from controls were significantly different from the H1N1-infected cells by Student’s *t* test (*** *p* < 0.001, ** *p* < 0.01). put-31 = put-miR-31; put-34 = put-miR-34; put-35 = put-miR-35.

**Figure 4:**
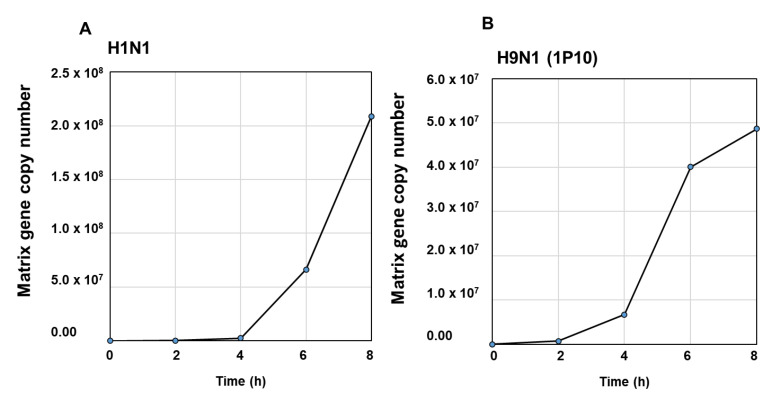
Viral copy numbers: IAV Matrix gene copy numbers analyzed using RT-PCR. (**A**) H1N1 and (**B**) H9N1(1P10).

To further evaluate the expression of put-miR-31, 34, and 35, HBEpCs were infected with H1N1 and H9N1(1P10) at MOI of 1.0, and RNA was isolated at different time intervals post infection (up to 8 h). The expression of put-miR-31 (Figure 2A) showed a decrease at 2 h and then gradually increased by 6 h followed by a reduction again after 8 h exposure to H1N1. In contrast, put-miR-34 (Figure 2B) showed a reduction in expression at all time points evaluated, although these values were not significant, but the expression pattern was similar to the NGS analysis of H1N1-infected cells. Put-miR-35 gradually increased in expression up to 4 h (Figure 2C) and returned to baseline level by 8 h. Interestingly, in H9N1(1P10)-infected cells, all three putative miRNAs showed significant downregulation at all time points studied and was in concordance with the data obtained in NGS analysis (Figure 3). In addition, put-miR-31 showed the highest downregulation (−1.3-fold) at 2 h (Figure 3A) and the downregulation of put-miR-34 was highest by 4 h (−2.1-fold) (Figure 3B). Put-miR-35 exhibited a gradual decrease in expression (Figure 3C) with increasing exposure time, and the highest (−2.4 fold) was at 8 h. Viral copy numbers in both strains increased with an increase in exposure time to the virus (Figure 4A,B).

### 3.3. STAT3 Expression Is Regulated by Put-miR-34.

**Figure 5:**
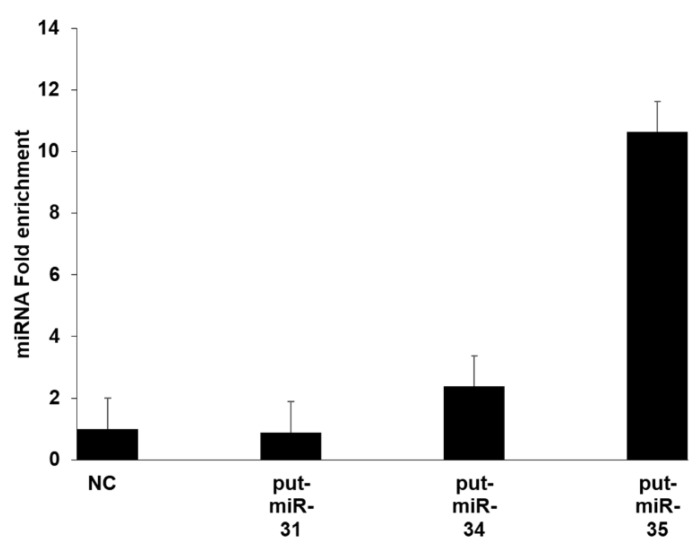
Efficiency of AGO immunoprecipitation in HBEpC transfected with put-miR-31, 34, 35 and a mimic control miRNA. miRNAs expression was normalized to the control that was set to 1. The fold enrichment was calculated based on the control transfected cells immunoprecipitated using both isotype and AGO antibodies. The results are representative of three independent experiments.

HBEpCs were transfected with the mimics of put-miR-31, 34, and 35 followed by AGO immunoprecipitation of total RNA from these cells. Put-miR-34 was enriched more than 2-fold and put-miR-35 was enriched 10-fold, while put-miR-31 had no significant change when compared to the scrambled miRNA control transfected cells (Figure 5).

**Figure 6:**
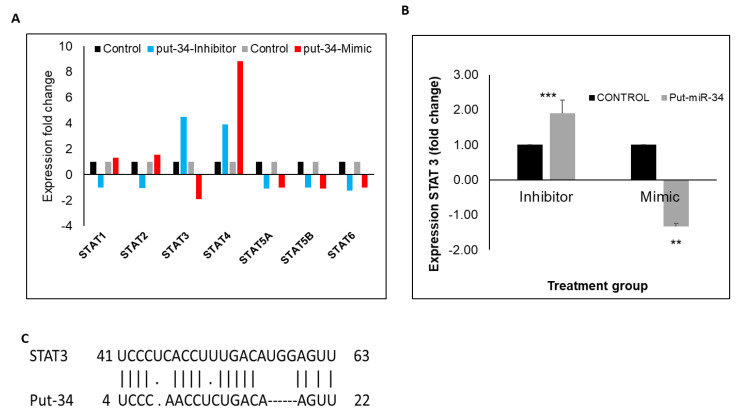
Put-miR-34 targets the STAT3 gene. (**A**) PCR array of transcription factors, and the STAT family protein data presented. (**B**) Put-miR-34 transfected with mimic and inhibitor showing the expression pattern of STAT3. (**C**) Homology of the put-miR-34 seed region to the UTR region of STAT3. The results of each control group were significantly different by Student’s *t* test ((*** *p* < 0.001, ** *p* < 0.01)). The results are representative of three independent experiments. put-34 = put-miR-34.

Since transcription factors can play a crucial role in regulating influenza infection, RNA from HBEpCs transfected with mimics or inhibitors of put-miR-31, 34, and 35 or their respective controls was assayed in a transcription factor RT-PCR array (84 transcription factors). Figure 6 shows the pattern of expression for the Signal transducers and activators of transcription (STAT) family of genes, where cells transfected with put-miR-34 inhibitor showed the highest upregulation of STAT3 and STAT4 transcripts. In addition, only cells transfected with put-miR-34 mimic showed a reduction in STAT3 expression, whereas STAT4 exhibited 9-fold upregulation (Figure 6A). The transcription factor array results were further confirmed using STAT3 specific Taqman primers (Figure 6B) in HBEpCs transfected with the mimic or inhibitor of put-miR-34. There was more than a 2-fold increase in STAT3 with inhibitor transfected HBEpCs and significant STAT3 reduction in mimic transfected cells. A homology search showed that the UTR region of STAT3 was 76% homologous to the seed region of the put-miR-34 (Figure 6C).

**Figure 7:**
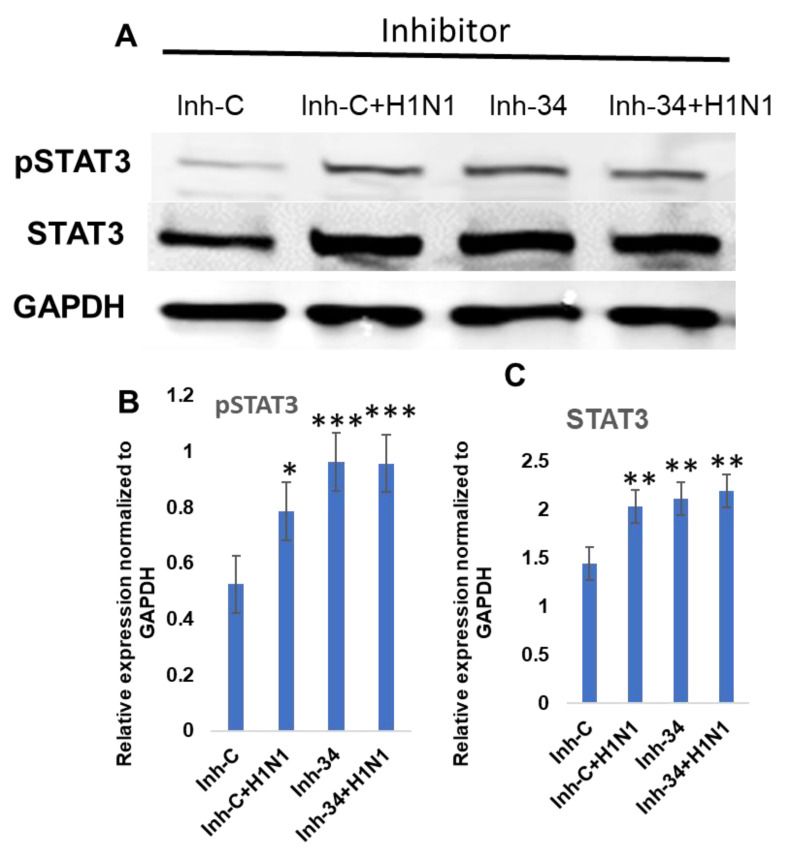
Immunoblot showing the overexpression of STAT3 and pSTAT3 in cells transfected with inhibitor of put-miR-34. (**A**) HBEpCs were transfected with inhibitor of put-miR-34 (Inh-34) or a scrambled miRNA inhibitor control (Inh-C) for 24 h and were subsequently infected with H1N1 of MOI of 1.0 for 18 h, the resulting cells were used for Western blot analysis. STAT3 was detected by Western blotting using a rabbit anti-STAT3 antibody and anti-pSTAT3 (Y705) antibody. Data representative of three biological replicates. (**B**) Quantification of pSTAT3 (Y705) normalized to GAPDH. (**C**) Quantification of STAT3 normalized to GAPDH. The results are representative of three independent experiments. The results from controls were significantly different from the treated by Student’s *t*-test. (*** *p* < 0.001, ** *p* < 0.01, * *p* < 0.05). Inh-C—Control scrambled miRNA, Inh-34—put-miR-34 inhibitor, pSTAT3—phosphorylated STAT3.

**Figure 8:**
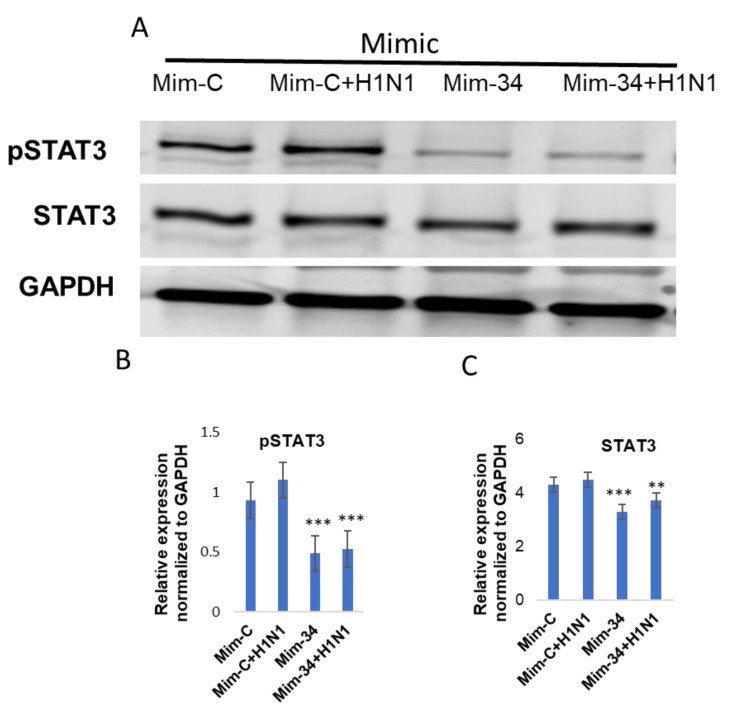
Immunoblot showing the abrogation of STAT3 in cells transfected with mimic of put-miR-34. (**A**) HBEpCs were transfected with a mimic of put-miR-34 and a scrambled miRNA mimic control for 24 h and were infected with an H1N1 MOI of 1 for another 18 h, the resulting cells were lysed, and protein was extracted for Western blot analysis. STAT3 was detected using a rabbit anti-STAT3 antibody and pSTAT3 by an anti-pSTAT3 (Y705) antibody. Data representative of three biological replicates. (**B**) Quantification of the pSTAT3 (Y705) normalized to GAPDH and (**C**) Quantification of the STAT3 normalized to GAPDH. The results from controls were significantly different from the put-miR-34 mimic transfected by Student’s *t*-test (*** *p* < 0.001, ** *p* < 0.01). Mim-C—Control scrambled mimics miRNA; Mim-34—put-miR-34 mimic, pSTAT3—phosphorylated STAT3.

When the cell lysate from put-miR-34 inhibitor transfected cells was tested for pSTAT3 and STAT3 by immunoblot, the inhibitor induced the expression of pSTAT3 and STAT3 proteins (Figure 7), and the level of expression of both pSTAT3 and STAT3 was significantly higher than in control cells transfected with scrambled miRNA. When the transfected cells were infected in H1N1, pSTAT3 increased in scrambled miRNA inhibitor control samples; however, the increase was significantly higher in the cells transfected with the put-miR-34 inhibitorIn addition, and we also observed a significant reduction in both STAT3 and pSTAT3 (>50%) in put-miR-34 mimic transfected cells (Figure 8). Although the H1N1 infection of control mimic transfected cells showed a moderate increase in pSTAT3, put-miR-34 mimic transfected cells infected with H1N1 did not induce the pSTAT3 (Figure 8). These data further confirm the role of STAT3 as a potential target of put-miR-34.

### 3.4. Functional Analysis of Put-miR-34

**Figure 9:**
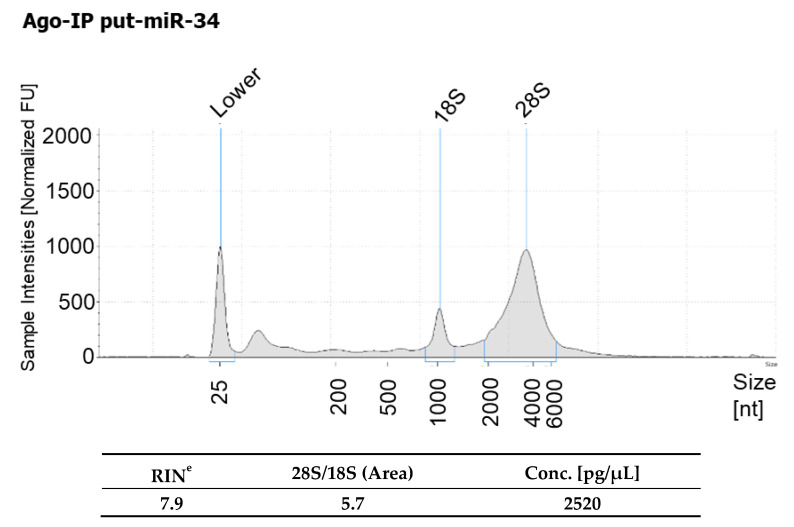
An overview of the RNA quality measured using Agilent Bioanalyzer of AGO immunoprecipitate of put-miR-34. RIN is the RNA integrity number.

To further confirm the data obtained as presented above, we transfected the HBEpCs with mimic of putative miRNAs (put-miR-31, put-miR-34, put-miR-35, and control) and repeated the AGO immunoprecipitation. The resulting RNA from AGO immunoprecipitation was subjected to NGS analysis to verify whether the transfected miRNA sequences can be retrieved from the complex. The data obtained from NGS, presented in Table 4, show the sequence count (raw data numbers). Interestingly, most of the predicted miRNAs were detected in the AGO immunoprecipitation, suggesting that these putative miRNAs were able to form a complex with the RISC complex. The scrambled control transfected cells did not show any significant increase in any of the putative miRNA sequences. The AGO immunoprecipitation RNA showed a good RNA integrity number (RIN), which was 7.9 (Figure 9).

GO analysis was performed on the data obtained from the NGS analysis of the AGO immunoprecipitation of these putative miRNAs and controls. In Table 5, the top 20 most significant GO terms associated with transcripts found to be differentially expressed between control mimics and mimic of put-miR-34 are given.

The GO terms associated with the differentially expressed transcripts were examined to see if certain biological functions are enriched among these transcripts compared to the reference. Interestingly, the GO term also predicted the modulation of transcription factors, confirming the possible implications of STAT3 transcription factor in regulating IAV replications and their modulation by put-miR-34.

## 4. Discussion

MiRNAs in humans and animals are triggered in response to environmental stimuli that include viral attack of the host. Several studies have shown that miRNA plays a critical role in viral replication and survival in host cells [6,7]. MiRBase is a database of all available miRNAs that are annotated with a name, and the majority of them have been studied in both human and animal models [1]. IAV infection induces several miRNAs, including both host and virus induced, although the specificity of the miRNAs is not well defined. It has become essential to explore the novel miRNAs elicited by IAV in humans that are directly or indirectly involved in IAV replication. There is a low degree of complementarity between the miRNA and its target sequence and that limits the computational target identification; thus, it has been essential to employ biochemical methods to verify the targets of miRNAs. Our NGS data from SAEpCs exposed to H1N1, H9N1(1P10), and H9N1(1WF10), predicted 42 novel miRNA (putative) sequences, and these sequences were further analyzed by RT-PCR. A time course study also revealed that put-miR-34 was differentially expressed in IAV-infected cells, and miRNA-34-targeted STAT3 mRNA. Mimic and inhibitor studies by PCR and Western blot analysis further confirmed that put-miR-34 targets STAT3. In addition, AGO immunoprecipitation analysis confirmed this as a potential miRNA candidate modulated during IAV infection and not a fragment of RNA degradation.

Once a candidate miRNA has been found, it becomes essential to identify its target(s) to understand the molecular mechanisms involved in viral replication. Indeed, as mentioned above, miRNAs bind to their targets with limited complementarity, and the effect of their binding on the target RNA expression is usually mild. Furthermore, several known target genes of miRNAs contain several miRNA-binding sites, and the degree of translational repression may increase exponentially with the number of miRNA binding sites in the 3′ untranslated region (UTR) [23]. We found that put-miR-34 is 76% homologous to the STAT3 UTR region. However, we have not determined whether this miRNA is highly specific for IAV strains or whether other viruses could modulate the expression of this miRNA.

Transcription factors are proteins that, through the action of binding to specific DNA sequences, control the transcription of genes. Transcription factors contain one or more DNA binding domain(s) that bind to either enhancer or promoter regions of DNA adjacent to the genes that they regulate. They have the function of either stabilizing (as an activator) or blocking (as a repressor) the binding of RNA polymerase to DNA and result in the direct control of gene regulation. As such, they are critical for various vital cellular development functions that include signal transduction, several signaling pathways (i.e., JAK/STAT, insulin, IGF, and others), and cell survival proteins. In this study, we identified novel miRNAs that are elicited following exposure to IAV, have a role in STAT signaling pathways, and may have a unique role in controlling the replication and survival of the virus in human bronchial epithelial cells.

STAT3 belongs to the family of STAT proteins, which are activated in response to extracellular signaling proteins, including the interleukin (IL)-6 family (IL-6, IL-11, leukemia inhibitory factor) [24]. In addition, cytokines utilizing the IL-6 signal-transducing receptor chain gp130 (IL-6) or homodimeric cytokine receptors (granulocyte colony-stimulating factor, G-CSF), as well as growth factors that act through protein tyrosine kinase receptors are involved in the activation of STAT3 [24,25,26,27]. STAT3 induces the expression of transcriptional repressors and co-repressors that inhibit NF-κB gene reporters [14,28]. The importance of transcription factor NF-κB in IAV replication is also known [6], suggesting an indirect mechanism by which STAT3 restrains pro-inflammatory gene transcription. The negative regulation of STAT3 by miR-20a via binding to the 3′-UTR of STAT3 mRNA was also demonstrated in pancreatic carcinoma cells [29]. We also have reported the increased expression of GCSF and IL-6 in influenza A and B patients’ serum [30]. In addition to these, put-miR-34 was recovered in AGO-immunoprecipitation complexes and from the mimic transfected cells (Table 4, Figure 5). GO terms predicted an increased modulation of several transcription factors, and that data corroborate our findings that put-miR-34 targets transcription factor STAT3. Upon phosphorylation, STAT3 translocates to the nucleus and induces the transcription of a large number of genes, including the STAT3 gene itself. IAV NS1 protein interferes with IFN production [31], which correlates to the reduced phosphorylation of STAT3. A recent study has shown that the H5N1 induced pSTAT3, which in turn delayed the apoptosis during IAV infection [32]. Consequently, a lower expression of STAT3 might be associated with reduced phosphorylation of STAT3, which eventually may affect some of the cell survival pathways. In addition, other studies have shown that IAV may have evolved a strategy to circumvent IL-6/STAT3-mediated immune response through upregulating SOCS3 [33]. STAT3 is also involved in the regulation of antiviral and pro-inflammatory responses [10] through the transcriptional regulation of other cellular factors or through pathways independent of its role as a transcription factor.

MiRNAs have become more and more attractive as diagnostic biomarkers and potential clinical intervention targets, as elective prevention and treatments for IAV infection is poorly available. In our previous studies, hsa-miR-548an was involved in the replication and survival of IAV by modulating the expression of NS1 A binding protein regulated through NF-κB [7]; similarly, miR-4776 targets NFKBIB protein [6], and miR-4276 regulates the COX6C protein [17] during IAV infections. These proteins are involved in the survival of the virus through NF-κB pathway or blocking the caspase-9-mediated apoptosis. The upregulation of microRNA-203 in influenza A virus infection inhibits viral replication by targeting the downregulator of transcription 1 [34]. In addition, miR-323, miR-491, and miR-654 inhibit replication of the H1N1 through binding to the PB1 gene [35]. miR-127-3p, miR-486-5p, and miR-593-5p were found to target at least one viral gene segment of both the human seasonal influenza H3N2 and PR8 (H1N1) [36]. Most of these studies were looking at miRNAs that target the viral genome, whereas our study was on a novel miRNA, put-miR-34, that targets host transcriptional protein STAT3 and modulates the phosphorylation during IAV infections.

Based on the unique features of miRNAs, a new generation of IAV vaccine may be developed by incorporating miRNA response elements (MRE, miRNA recognition sequences) into viral genomic segments such as NP, NS, or PB1. Furthermore, the direct targeting of key miRNAs that underpin IAV infection may lead to new and more specific therapeutic interventions, as these small RNAs are implicated in regulating cytokines induced by infection. It is necessary to explore novel miRNAs that can both degrade IAV RNAs and ease virus-induced infection, as they may concurrently control both virus replication and over-reactive immune responses.

## 5. Conclusions

In conclusion, the present study examined the differential expression of put-miR-34 in airway epithelial cells during IAV infections. The results provide new evidence that a novel put-miR-34 is strongly linked to high STAT3 regulation. These observations are consistent with the collection of evidence implicating crucial roles for put-miR-34 in regulating viral responses in cell systems. Our current results demonstrate that put-miR-34 is relevant to the immunobiology of the human airways and highlight the need to conduct additional studies to better define the physiological role of put-miR-34 in the respiratory tract of humans.

## Figures and Tables

**Table 1:** Summary of the predicted miRNA. Putative novel microRNAs were predicted from the sequences that do not map to any organism found in miRbase or other known RNA sequences. miRPara was used to analyze the potential folding of these sequences, and the results were combined to identify the putative novel miRNAs.

Putative miRNA	Chromosome	Strand	Start	Stop	Sequence
put-miR-1	10	+	64,075,172	64,075,189	GAGTGTGAGTCTGAAACT
put-miR-2	12	+	12,227,225	12,227,242	GCCCGGATAGCTCAGTCA
put-miR-3	14	+	103,804,182	103,804,209	TTGCAAGCAACACTCTGTGGCAGATGAT
put-miR-4	1	+	28,880,952	28,880,969	ATTACGATCTGCTGAGTA
put-miR-5	1	+	90,118,586	90,118,605	GAGAGGGTGCTGTAGGCTCA
put-miR-6	1	+	145,951,004	145,951,032	AGTTCAGTGGTAGAATTCTCGCCTCCCAC
put-miR-7	1	+	147,492,721	147,492,750	CAGTTCAGTGGTAGAATTCTCGCCTCCCAC
put-miR-8	1	+	161,582,560	161,582,588	TTCGATTCCCGGGTAACGAAACGTTTTTG
put-miR-9	1	+	183,212,789	183,212,813	GAATAGACTGGATGGAAAGACAAAC
put-miR-10	1	+	183,212,907	183,212,930	AATGTGACTAAAGGAAAAAACTTT
put-miR-11	1	+	183,213,617	183,213,638	TGACATAGTCTCTGCCCTCATA
put-miR-12	20	+	3,194,760	3,194,784	GCAAAATGATGAGGTACCTGATACT
put-miR-13	20	+	18,309,660	18,309,685	ATGGTAGTGGGTTATCAGAACTTATT
put-miR-14	20	+	32,082,877	32,082,893	TTGCTCTGATGAAATCT
put-miR-15	2	+	164,382,863	164,382,879	ACAGTGACTGAGAGACT
put-miR-16	3	+	164,059,153	164,059,174	TATCTCGCTGGGGCCTCCAAAA
put-miR-17	4	+	83,354,324	83,354,340	ACTACCGTTTTCTGAAG
put-miR-18	5	+	105,889,151	105,889,173	GTTCTTGTAGTTGAAATACAACG
put-miR-19	5	+	105,889,191	105,889,208	TGGTCGTGGTTGTAGTCC
put-miR-20	5	+	180,528,918	180,528,945	GCCAGCTTGTTGTGATTCCTCCATTTTT
put-miR-21	6	+	11,976,724	11,976,740	TTTCCTTCTGAGAACAA
put-miR-22	6	+	27,656,041	27,656,059	AGTATTCTCTGTGGCTTTT
put-miR-23	6	+	28,574,986	28,575,007	TCAATCCCCGGCACCTCCACCA
put-miR-24	7	+	18,159,276	18,159,293	AAATCTGACTGTCTAATT
put-miR-25	7	+	123,501,242	123,501,257	AGTTTCTGTCTGATAA
put-miR-26	7	+	139,025,502	139,025,531	GAGTCCCATCTGGGGTGGCCTGTGACTTTT
put-miR-27	9	+	122,841,421	122,841,437	TAGCAAGACTGAGGCTT
put-miR-28	9	+	133,282,156	133,282,176	AGCCTGTCTGAGCGCCGCTCT
put-miR-29	18	+	19,645,301	19,645,318	AAAGAAGTTCTGAGCTTG
put-miR-30	18	+	68,180,033	68,180,063	CAGGAGTTCTGGGCTGTAGTGCGCTATGCT
put-miR-31	22	+	35,126,604	35,126,623	CCAACTTCCTTCTGAGAACA
put-miR-32	15	+	45,009,923	45,009,945	ACATGGACATGATCTTCTTTATA
put-miR-33	MT	−	16,525	16,551	AAGGGGAACGTGTGGGCTATTTAGGCT
put-miR-34	X	+	23,803,909	23,803,934	AAGAGGGTTGGAGACTGTTCAAGATC
put-miR-35	X	+	34,234,100	34,234,123	GAGCAGTAACAGGTCTGTGATGCT
put-miR-36	8	+	100,305,233	100,305,261	GTTACTAGAGAAGTTTCTCTGAACGTGTA
put-miR-37	16	+	2,737,223	2,737,253	CTCTGGTGATGAAATGGAACGTTTCTGATG
put-miR-38	16	+	33,963,846	33,963,873	TTGGTGGAGTGATTTGTCTGGTTAATTC
put-miR-39	16	+	33,963,882	33,963,908	AACGAGACTCTGGCATGCTAACTAGTT
put-miR-40	11	+	49,764,393	49,764,415	TATCCCGGACGAGCCCCCATTAT
put-miR-41	11	+	62,126,505	62,126,531	TGGTGTAATGGTTAGCACTCTGGACTC
put-miR-42	11	+	65,271,895	65,271,916	GTGAAACGACTGGAGTATGATT

**Table 2:** Data on the RT-PCR of the predicted miRNAs for H1N1. HBEpCs were exposed to H1N1 for 3 h, and the miRNA were extracted and analyzed for the expression of putative miRNAs. The fold change, *p*-value, and the Benjamini–Hochberg false discovery rate (FDR) values are shown. Data ranked based on the *p*-value. Not all miRNAs were detected in RT-PCR analysis. ND—not detected.

Putative miRNA	Fold Change H1N1 /Mock	*t*-Test *p*-Value	Benjamini– Hochberg FDR
put-miR-34	−2.725	0.013	0.315
put-miR-18	−1.823	0.098	0.675
put-miR-26	−1.652	0.124	0.675
put-miR-36	−2.338	0.143	0.675
put-miR-22	1.494	0.172	0.675
put-miR-31	1.636	0.213	0.675
put-miR-28	−1.741	0.227	0.675
put-miR-7	1.744	0.241	0.675
put-miR-19	−2.057	0.272	0.675
put-miR-13	−1.501	0.283	0.675
put-miR-39	−1.490	0.338	0.675
put-miR-20	−1.184	0.366	0.675
put-miR-38	−1.447	0.397	0.675
put-miR-41	−1.699	0.465	0.675
put-miR-23	1.163	0.471	0.675
put-miR-30	1.256	0.540	0.675
put-miR-35	1.230	0.625	0.744
put-miR-5	1.247	0.738	0.802
put-miR-8	−1.173	0.826	0.860
put-miR-1	1.027	0.958	0.958
put-miR-10	3.130	ND	ND
put-miR-11	−1.080	ND	ND
put-miR-27	1.207	ND	ND
put-miR-29	2.776	ND	ND
put-miR-37	−1.147	ND	ND
put-miR-40	−1.079	ND	ND
put-miR-6	1.524	ND	ND

**Table 3:** RT-PCR data on the predicted miRNAs for H9N1 (1P10). HBEpC exposed to H9N1 for 3 h, and the miRNA were extracted and analyzed for the expression of putative miRNAs. The fold change, *p*-value, and the Benjamini–Hochberg false discovery rate (FDR) values are shown. Data ranked based on the *p*-value. Not all miRNAs were detected in RT-PCR analysis. ND—not detected.

Putative miRNA	Fold Change H9N1-IP10/Mock	*t*-Test *p*-Value	Benjamini–Hochberg FDR
put-miR-31	2.213	0.050	0.675
put-miR-34	−3.009	0.067	0.866
put-miR-23	−1.211	0.192	0.989
put-miR-22	−1.237	0.205	0.989
put-miR-41	1.718	0.293	0.989
put-miR-18	−1.377	0.323	0.989
put-miR-26	−1.413	0.454	0.989
put-miR-39	−1.056	0.503	0.989
put-miR-35	−1.312	0.525	0.989
put-miR-20	−1.452	0.590	0.989
put-miR-1	−1.471	0.593	0.989
put-miR-30	1.149	0.656	0.989
put-miR-36	−1.378	0.673	0.989
put-miR-8	−1.229	0.686	0.989
put-miR-7	1.146	0.710	0.989
put-miR-28	1.101	0.768	0.989
put-miR-19	−1.087	0.794	0.989
put-miR-6	1.221	0.802	0.989
put-miR-5	−1.024	0.948	0.989
put-miR-13	1.006	0.985	0.989
put-miR-38	−1.007	0.989	0.989
put-miR-10	1.105	ND	ND
put-miR-11	−1.016	ND	ND
put-miR-27	1.691	ND	ND
put-miR-29	1.090	ND	ND
put-miR-37	−1.086	ND	ND
put-miR-40	−1.286	ND	ND

**Table 4:** Recovered putative miRNA counts using NGS. HBEpCs were transfected with miRNA mimics of putative miRNAs, the cell lysates were immunoprecipitated with AGO antibodies, and the isolated RNA was subjected to NGS analysis; the resulting counts of the specific putative miRNAs recovered (raw data numbers) are presented. IP: Argonaute immunoprecipitated.

Raw Data Counts				
Ago-IP	Put-miR-31	Put-miR-34	Put-miR-35	Control-SCR
Put-miR-31	29,275	1949	681	3866
Put-miR-34	221	117,229	901	422
Put-miR-35	115	138	263,227	61

**Table 5:** Mimics of put-miR-34 transfected cells analyzed using NGS show the top 20 most significant GO terms associated with transcripts found to be differentially expressed between control mimics and mimic of put-miR-34. This table highlights the most relevant GO terms associated with the differentially expressed transcripts in comparison to see if certain biological functions are enriched among these transcripts compared to the reference background. This analysis is a type of gene enrichment test and does not ensure that the transcripts that belong to a significant GO term are upregulated or downregulated. However, it ensures that a group of differentially expressed genes with similar functionality are significantly overrepresented. The expected values represent an estimate of the number of transcripts associated with the given GO term that are significant by random among all the annotated differentially expressed genes. The significant (observed) values represent the number of differentially expressed transcripts associated with that GO term in the sample (real) dataset. Annotations represents the total number of genes associated with that GO term in the sample dataset, which means that the reference background could potentially have higher number of annotations. The *p*-value represents the test statistics for the given GO term whether it is significantly enriched or not.

GO_ID	Term	Annotated	Significant	Expected	*p*-Value
GO:0006281	DNA repair	427	5	5.07	5.1 × 10^−14^
GO:0019048	modulation by virus of host morphology or physiology	336	8	3.99	2.1 × 10^−11^
GO:0007067	mitotic nuclear division	359	4	4.26	6.9 × 10^−10^
GO:0051301	cell division	577	6	6.85	1.4 × 10^−09^
GO:0006886	intracellular protein transport	821	8	9.75	6.3 × 10^−09^
GO:0000398	mRNA splicing, via spliceosome	210	3	2.49	6.4 × 10^−08^
GO:0006355	regulation of transcription, DNA templated	3012	37	35.78	6.6 × 10^−08^
GO:0043123	positive regulation of I-kB kinase/NF-κB signaling	153	6	1.82	8.6 × 10^−08^
GO:0016197	endosomal transport	152	2	1.81	2.0 × 10^−07^
GO:0000209	protein polyubiquitination	163	0	1.94	2.7 × 10^−07^
GO:0000184	nuclear-transcribed mRNA catabolic process, nonsense-mediated decay	109	0	1.29	2.8 × 10^−07^
GO:0006364	rRNA processing	114	1	1.35	3.0 × 10^−07^
GO:0000086	G2/M transition of mitotic cell cycle	158	2	1.88	4.2 × 10^−07^
GO:0000082	G1/S transition of mitotic cell cycle	223	0	2.65	1.5 × 10^−06^
GO:0006614	SRP-dependent co-translational protein targeting to membrane	101	1	1.2	1.5 × 10^−06^
GO:0010467	gene expression	4410	49	52.38	2.5 × 10^−06^
GO:0006413	translational initiation	156	3	1.85	2.6 × 10^−06^
GO:0006415	translational termination	86	0	1.02	3.3 × 10^−06^
GO:0034138	toll-like receptor 3 signaling pathway	80	1	0.95	3.5 × 10^−06^
GO:0051726	regulation of cell cycle	701	7	8.33	4.1 × 10^−06^

## Data Availability

Not applicable.
